# Enhanced Antitumor Efficacy of Radium-223 and Enzalutamide in the Intratibial LNCaP Prostate Cancer Model

**DOI:** 10.3390/ijms24032189

**Published:** 2023-01-22

**Authors:** Mari I. Suominen, Matias Knuuttila, Christoph A. Schatz, Andreas Schlicker, Jukka Vääräniemi, Birgitta Sjöholm, Esa Alhoniemi, Bernard Haendler, Dominik Mumberg, Sanna-Maria Käkönen, Arne Scholz

**Affiliations:** 1 Pharmatest Services Ltd., 20520 Turku, Finland; 2 Aurexel Life Sciences Ltd., 21240 Askainen, Finland; 3 Research & Development, Pharmaceuticals, Bayer AG, 13353 Berlin, Germany; 4 Inoi Oy, 20100 Turku, Finland; 5 Institute of Biomedicine, University of Turku, 20520 Turku, Finland

**Keywords:** radium-223, enzalutamide, prostate cancer, targeted alpha therapy, bone metastases, CRPC

## Abstract

Radium-223 dichloride and enzalutamide are indicated for metastatic castration-resistant prostate cancer and their combination is currently being investigated in a large phase 3 clinical trial. Here, we evaluated the antitumor efficacy of radium-223, enzalutamide, and their combination in the intratibial LNCaP model mimicking prostate cancer metastasized to bone. *In vitro* experiments revealed that the combination of radium-223 and enzalutamide inhibited LNCaP cell proliferation and showed synergistic efficacy. The combination of radium-223 and enzalutamide also demonstrated enhanced *in vivo* antitumor efficacy, as determined by measuring serum PSA levels in the intratibial LNCaP model. A decreasing trend in the total area of tumor-induced abnormal bone was associated with the combination treatment. The serum levels of the bone formation marker PINP and the bone resorption marker CTX-I were lowest in the combination treatment group and markedly decreased compared with vehicle group. Concurrent administration of enzalutamide did not impair radium-223 uptake in tumor-bearing bone or the ability of radium-223 to inhibit tumor-induced abnormal bone formation. In conclusion, combination treatment with radium-223 and enzalutamide demonstrated enhanced antitumor efficacy without compromising the integrity of healthy bone. The results support the ongoing phase 3 trial of this combination.

## 1. Introduction

Radium-223 dichloride (radium-223) is the first and only targeted alpha therapy used for the treatment of patients with metastatic castration-resistant prostate cancer (mCRPC). Radium-223 has been shown to improve overall survival and quality of life and reduce symptomatic skeletal events (SSEs) in patients with mCRPC [1,2]. Due to its calcium-mimicking properties, radium-223 binds to newly-formed bone matrix, particularly in areas of active bone turnover, such as bone metastases. Once incorporated into bone matrix, radium-223 induces DNA double-strand breaks in cancer cells, osteoblasts, and osteoclasts via alpha radiation [3].

Enzalutamide is a second-generation androgen receptor (AR) inhibitor used for the treatment of men with mCRPC [4], metastatic hormone-sensitive prostate cancer [5], or non-metastatic CRPC [6,7]. Pivotal phase 3 trials AFFIRM and PREVAIL have confirmed the antitumor efficacy of enzalutamide in mCRPC patients with bone metastases [4,8,9]. Due to the distinctly different mechanisms of action of radium-223 and enzalutamide, combination treatment holds potential for improving the outcomes of patients with mCRPC. Enzalutamide has been shown to radiosensitize cancer cells to radiation therapy *in vitro* by affecting the DNA damage repair pathway [10] and the expression of inflammation- and metabolism-related genes [11], and to enhance radiation-induced antitumor efficacy in mice bearing LNCaP tumors [12]. Promising results suggesting favorable safety and tolerability of the radium-223 and enzalutamide combination treatment have been reported recently [13,14]. Currently, the combination of radium-223 and enzalutamide is under investigation in the clinical phase 3 PEACE III (EORTC-1333-GUCG) trial [15,16].

Here, we report the preclinical characterization of radium-223 in combination with the AR inhibitor enzalutamide. We evaluated the antitumor efficacy of radium-223 treatment and its possible effects on bone in combination with enzalutamide in the intratibial LNCaP xenograft model mimicking prostate cancer metastasized to bone. Taken together, the results support the ongoing clinical development of radium-223 in combination with enzalutamide for the treatment of mCRPC.

## 2. Results

### 2.1. The Combination of Radium-223 and Enzalutamide Has Synergistic Antiproliferative Efficacy In Vitro

**Figure 1:**
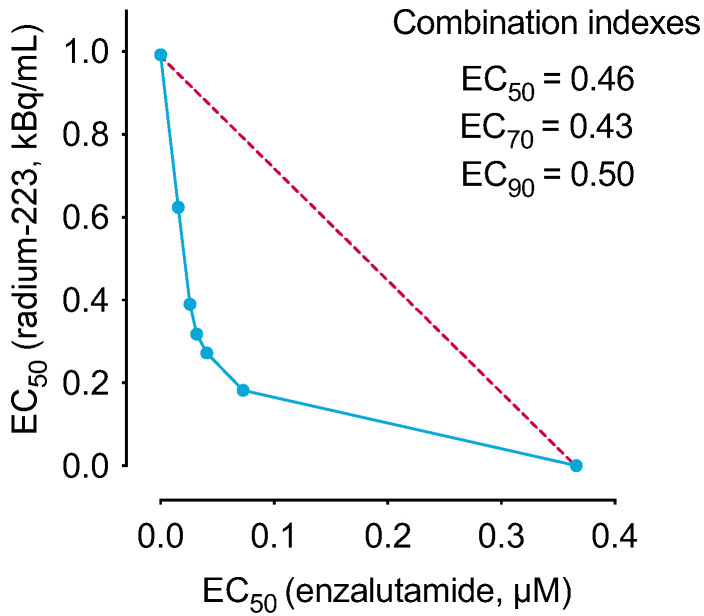
The combination of radium-223 and enzalutamide has synergistic antiproliferative efficacy *in vitro*. Isobologram for the *in vitro* combination effect of radium-223 and enzalutamide on the cell viability of LNCaP prostate cancer cells. Combination indexes were calculated for the combination treatment of radium-223 and enzalutamide with 0.3–0.7 defined as synergism and 0.70–0.85 defined as moderate synergism.

Cell viability of androgen-sensitive LNCaP cells was assessed by exposing the cells to radium-223 and enzalutamide treatment in androgen-depleted conditions *in vitro*. The combination of radium-223 and enzalutamide showed a synergistic antiproliferative effect with combination indexes between 0.43–0.50 (Figure 1). The single compound EC_50_ values for radium-223 and enzalutamide were 1.03 kBq/mL and 3.71 µM, respectively.

### 2.2. Enzalutamide Treatment Downregulates Pathways Involved in DNA Damage Repair

**Figure 2:**
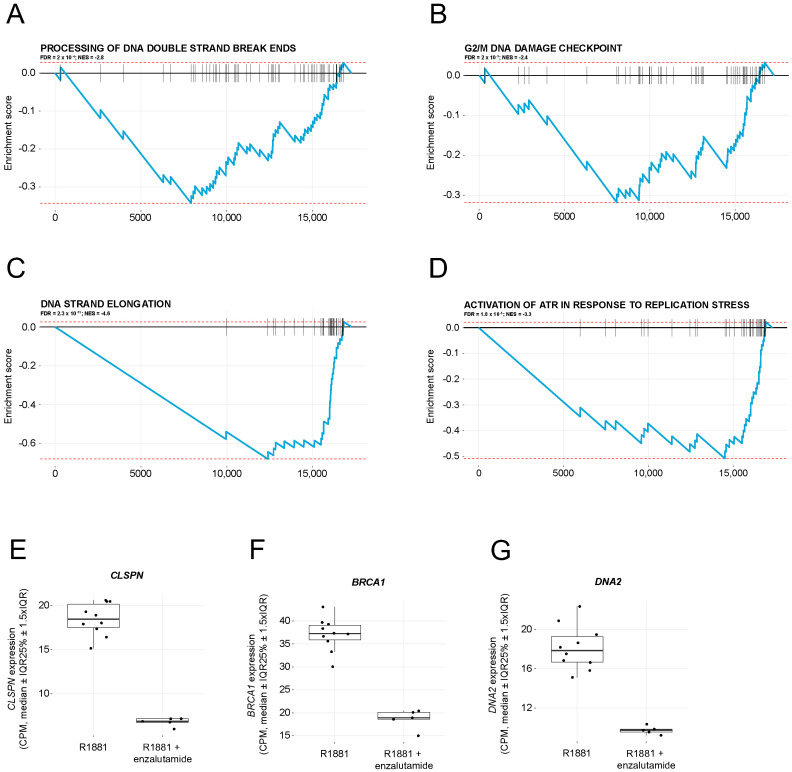
Enzalutamide treatment downregulates pathways involved in DNA damage repair. Gene set enrichment analysis (GSEA) of downregulated pathways related to DNA damage repair in LNCaP cells after treatment with enzalutamide. Enrichment plots for the Reactome pathways: (**A**) “Processing of DNA double-strand breaks ends”, (**B**) “G2/M DNA damage checkpoint”, (**C**) “DNA strand elongation”, and (**D**) “Activation of ATR in response to replication stress”. Effect size and statistical significance are indicated by normalized enrichment scores (NES) and false discovery rate (FDR) for each pathway. Gene expression of (**E**) *CLSPN*, (**F**) *BRCA1*, and (**G**) *DNA2* for samples stimulated with R1881 or treated with enzalutamide after R1881 stimulation. Boxplots describe counts per million (CPM, median ± IQR25% ± 1.5xIQR).

The expression of DNA damage repair genes was studied in LNCaP cells treated with the synthetic androgen R1881 or the combination of R1881 and enzalutamide. Gene set enrichment analysis (GSEA) of RNA sequencing data showed downregulation of four pathways involved in DNA damage response (Figure 2A–D). Notably, mRNA expression of genes contributing to the processing of DNA double-strand break ends (Figure 2A) and the G2/M DNA damage checkpoint (Figure 2B) was downregulated after enzalutamide treatment. On a single gene level, *CLSPN*, *BRCA1*, and *DNA2* (Figure 2E–G), all involved in DNA damage repair signaling, were prominently downregulated by the enzalutamide treatment, from almost 50% up to a 63% decrease when compared with R1881 stimulation without enzalutamide.

### 2.3. Combination Treatment with Radium-223 and Enzalutamide Exhibits Enhanced Antitumor Efficacy In Vivo

**Figure 3:**
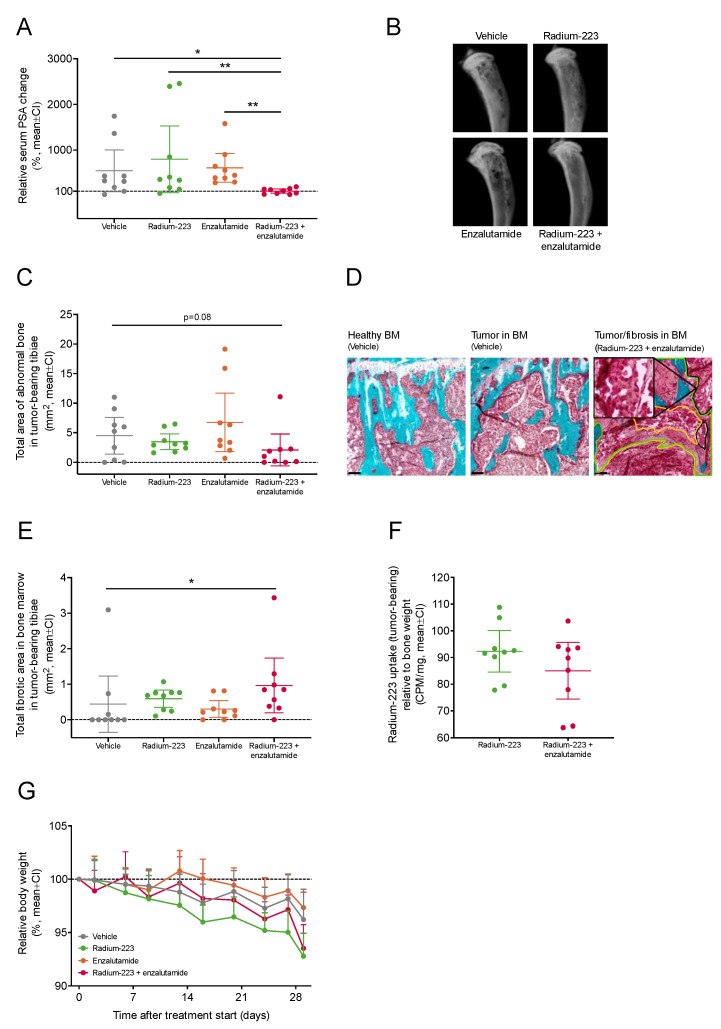
Combination treatment with radium-223 and enzalutamide exhibits enhanced antitumor efficacy *in vivo*. Blood samples were collected from the saphenous vein of mice (*n* = 9/group) treated with vehicle, radium-223 (330 kBq/kg, Q4W × 2), enzalutamide (30 mg/kg, QD, p.o.) or the combination of radium-223 and enzalutamide. (**A**) Serum PSA change at the end of the study relative to the pre-treatment baseline. (**B**) Representative *ex vivo* X-ray images of tumor-bearing tibiae. (**C**) Total area of abnormal bone measured by radiography. (**D**) Representative histology images (10×) of healthy bone marrow (BM) and LNCaP tumor tissue in bone marrow stained with Masson-Goldner’s trichrome. Tumor (black lines, with a zoomed insert of tumor), necrotic (yellow lines) and fibrotic (green lines) tissue areas are annotated in the image on right. Scale bar length: 100 μm. (**E**) Total fibrotic area in the bone marrow of tumor-bearing tibiae as determined by histology. (**F**) Radium-223 uptake in bone determined by measuring radioactivity in the tumor-bearing tibiae. The results are expressed as counts per minute (CPM) normalized to the weight of the bone sample. Plots describe mean and 95% confidence interval (CI). (**G**) Relative body weights of mice treated with vehicle, radium-223, enzalutamide, or their combination. Body weights were recorded twice weekly during the treatment period The figure describes relative body weight compared to the treatment start, shown as mean and 95% confidence interval (CI). The statistical analyses were performed using ANOVA (**A**,**C**), Kruskal–Wallis test and pairwise comparisons using Dunn’s test (**E**), or Welch’s *t*-test (**F**): * *p* < 0.05; ** *p* < 0.01.

The antitumor efficacy of the radium-223 and enzalutamide combination treatment was evaluated in the intratibial LNCaP xenograft mouse model that mimics prostate cancer metastasized to bone. Of all inoculated mice, 48% showed established tumor growth with prostate-specific antigen (PSA) levels over 0.12 ng/mL six weeks after inoculation. At the start of treatment, the mean PSA value was 1.57 ng/mL (SD 2.04 ng/mL, range 0.12–11.9 ng/mL). The mice were administered with vehicle, radium-223 (330 kBq/kg, on days 0 and 28), enzalutamide (30 mg/kg, once daily for 28 days), or with a combination. In the radium-223 or enzalutamide monotherapy groups, the relative PSA change from the pre-treatment baseline did not differ from the vehicle group, but the radium-223 and enzalutamide combination treatment completely prevented the PSA increase (Figure 3A). Furthermore, the relative change from the baseline serum PSA value was significantly lower in the combination group when compared with vehicle (*p* = 0.04), radium-223 (*p* = 0.008) or enzalutamide (*p* = 0.002) monotherapy groups (Figure 3A). In addition, a statistical interaction between radium-223 and enzalutamide treatments was found (*p* = 0.003), confirming the synergistic effect on serum PSA. A decreasing trend (*p* = 0.08; 46% of vehicle) in the total area of abnormal bone was observed with the combination treatment when compared with vehicle (Figure 3B,C). As expected, intratibially inoculated LNCaP cells formed tumors within the bone marrow (Figure 3D). Upon combination treatment, the total fibrotic area detected in the bone marrow increased (*p* = 0.03) when compared with vehicle (Figure 3E). The fibrotic areas were found in the vicinity of tumors and could have been a result of tumor tissue eradication due to combination treatment (Figure 3D). The second injection of radium-223 was given on day 28, one day before sacrifice, to measure the uptake of radium-223 in bone. Compared with radium-223 monotherapy, concurrent enzalutamide treatment did not affect the radium-223 uptake in tumor-bearing (Figure 3F). No marked body weight loss (>10%) was observed in any of the treatment groups (Figure 3G), demonstrating that all treatments were well tolerated.

### 2.4. Radium-223 and Enzalutamide Combination Treatment Inhibits Abnormal Bone Turnover

**Figure 4:**
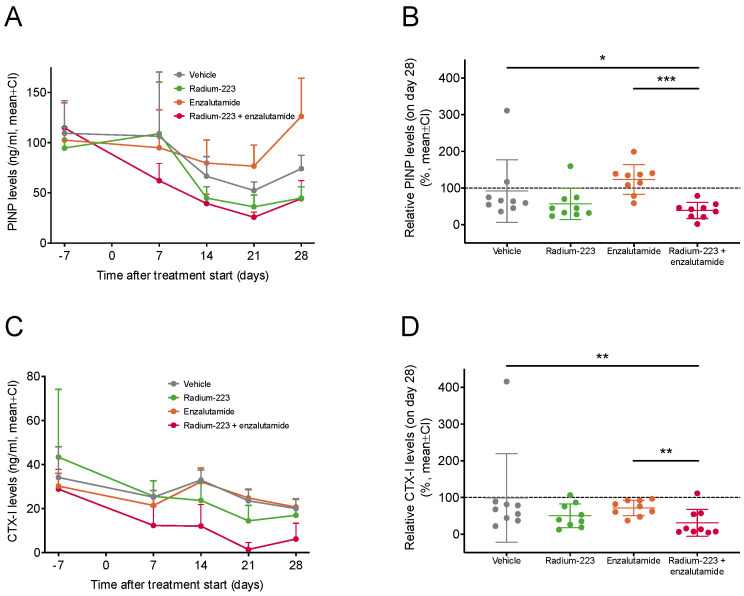
Radium-223 and enzalutamide combination treatment inhibits abnormal bone turnover. (**A**,**B**) PINP and (**C**,**D**) CTX-I bone marker levels were measured in blood samples collected from the saphenous vein of mice (*n* = 9/group) 7 days prior to and 7, 14, 21, and 28 days after the initiation of treatment. The mice were treated with vehicle, radium-223 (330 kBq/kg, Q4W × 2), enzalutamide (30 mg/kg, QD, p.o.) or the combination of radium-223 and enzalutamide. Values describe mean and 95% confidence interval (CI). The statistical analyses were performed using mixed models and model contrasts: * *p* < 0.05; ** *p* < 0.01; *** *p* < 0.001.

To evaluate the effects of radium-223 and enzalutamide on bone turnover, serum levels of the bone formation marker procollagen type I N-terminal propeptide (PINP) and the bone resorption marker C-terminal telopeptide of type I collagen (CTX-I) were measured. A prominent decline in PINP levels was observed in mice treated with the combination of radium-223 and enzalutamide (Figure 4A). At the end of the study, PINP levels were significantly lower in mice administered with the combination treatment than in vehicle-treated (*p* = 0.03) or enzalutamide-treated (*p* < 0.001) mice, while no difference in comparison to radium-223-treated mice was observed (Figure 4B). Correspondingly, CTX-I levels decreased in mice treated with the combination treatment (Figure 4C), resulting in lower CTX-I levels compared with the vehicle (*p* = 0.004) and enzalutamide monotherapy (*p* = 0.004) (Figure 4D). Taken together, these data suggest that the combination treatment with radium-223 and enzalutamide inhibits tumor-induced abnormal bone remodeling.

### 2.5. Concurrent Enzalutamide Administration Does Not Impair Radium-223-Specific Antitumor Effects in Bone or Its Ability to Inhibit Abnormal Bone Formation

**Figure 5:**
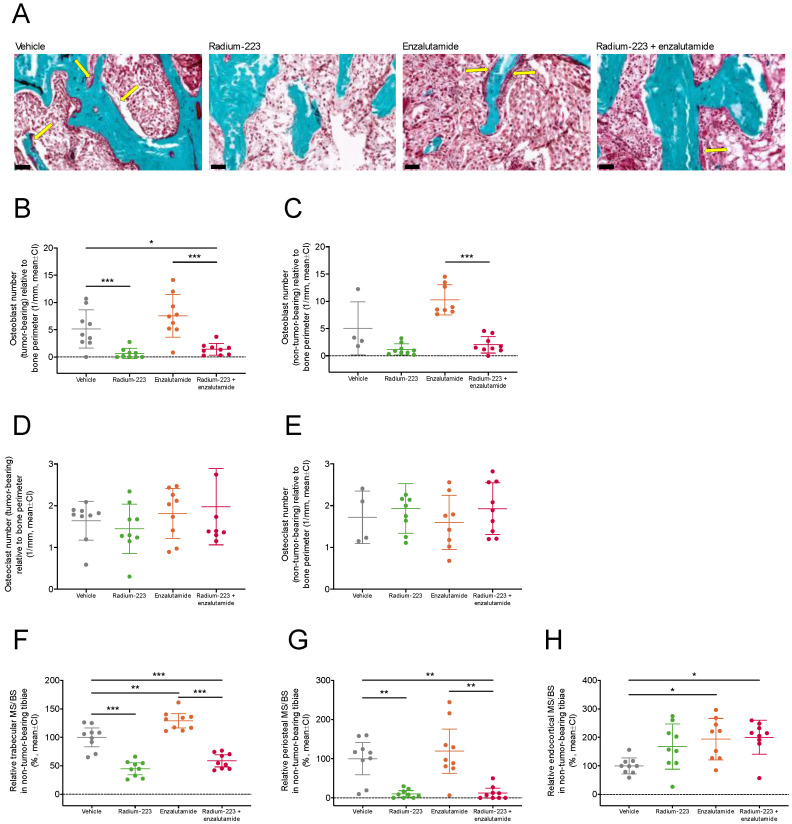
Concurrent enzalutamide administration does not impair radium-223-specific antitumor effects in bone or its ability to inhibit abnormal bone formation. The mice (*n* = 9/group) were treated with vehicle, radium-223 (330 kBq/kg, Q4W × 2, i.v.), enzalutamide (30 mg/kg, QD, p.o.), or their combination. The bones were labeled with alizarin red and calcein green *in vivo* for dynamic histomorphometry. (**A**) Representative images (20×) of osteoblast clusters (indicated with yellow arrows) in tumor-bearing tibiae in all treatment groups visualized by Masson-Goldner’s trichrome staining. Scale bar length: 50 μm. The number of (**B**,**C**) osteoblasts and (**D**,**E**) osteoclasts relative to bone perimeter in tumor-bearing (*n* = 9/group) and non-tumor-bearing tibiae (*n* = 9/group, except vehicle, *n* = 4; enzalutamide, *n* = 8) as determined by histomorphometry. Bone formation parameters, (**F**) trabecular, (**G**) periosteal and (**H**) endocortical mineralizing surface per trabecular, periosteal and endocortical bone surface (MS/BS), respectively, were measured in the non-tumor-bearing tibiae by dynamic histomorphometry. Plots indicate mean and 95% confidence interval (CI). The statistical analyses were performed using ANOVA followed by contrasts, or Kruskal–Wallis test followed by Dunn’s test: * *p* < 0.05; ** *p* < 0.01; *** *p* < 0.001.

To further evaluate the effects of concurrent enzalutamide treatment on radium-223 activity, the number of osteoclasts and disease-driving osteoblasts in tumor-bearing and non-tumor-bearing tibiae were quantified. The relative number of osteoblasts in the tumor-bearing tibiae was reduced by radium-223 monotherapy (*p* < 0.001) and the combination treatment (*p* = 0.04) compared with vehicle (Figure 5A,B), while the reduction upon combination treatment did not reach statistical significance in the non-tumor-bearing bone (Figure 5C). In contrast, the number of osteoclasts in tumor-bearing (Figure 5D) or non-tumor-bearing (Figure 5E) tibiae was not affected by any of the treatments. The amount of trabecular (Figure 5F) and periosteal (Figure 5G) mineralizing surface were decreased by radium-223 alone and in combination with enzalutamide compared with vehicle. No changes were observed in the endocortical mineralizing surface between the combination therapy and either of the monotherapies (Figure 5H). Together, these data demonstrate that antitumor efficacy of radium-223 and its ability to inhibit abnormal bone formation were not affected by concurrent enzalutamide administration.

### 2.6. Radium-223 and Enzalutamide Combination Treatment Does Not Affect Bone Microarchitecture

**Figure 6:**
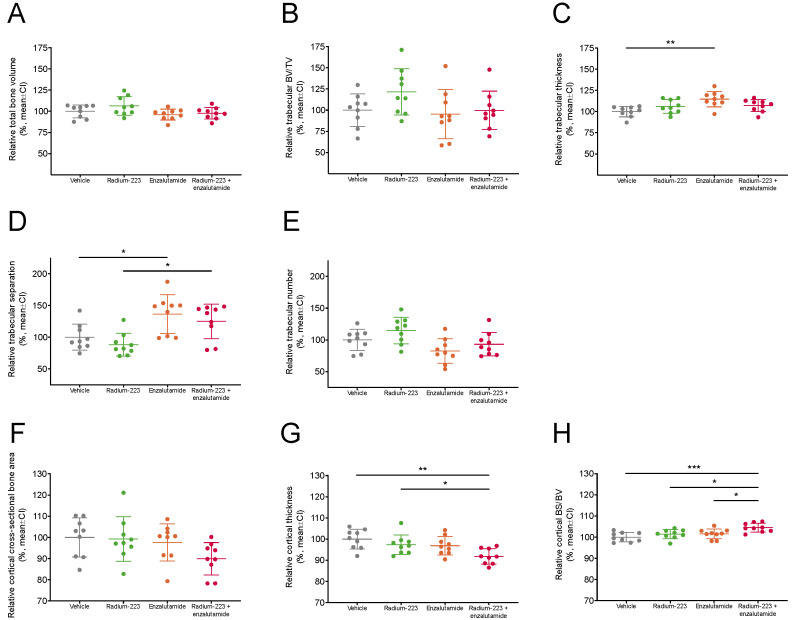
Radium-223 and enzalutamide combination treatment does not affect bone microarchitecture. The mice (*n* = 9/group) were treated with vehicle, radium-223 (330 kBq/kg, Q4W × 2, i.v.), enzalutamide (30 mg/kg, QD, p.o.), or their combination. Bone structure parameters (relative to vehicle) described as (**A**) total bone volume (BV), (**B**) trabecular bone volume fraction (BV/TV), (**C**) trabecular thickness, (**D**) trabecular separation, (**E**) trabecular number, (**F**) cortical cross-sectional bone area, (**G**) cortical thickness, and (**H**) cortical bone specific surface (BS/BV). Plots indicate mean and 95% confidence interval (CI). The statistical analyses were performed using ANOVA followed by contrasts or Kruskal–Wallis test followed by Dunn’s test: * *p* < 0.05; ** *p* < 0.01; *** *p* < 0.001.

To investigate whether the combination of radium-223 and enzalutamide affects bone quality and structure, densitometric and morphometric measures of total, trabecular, and cortical bone compartments were assessed in non-tumor-bearing tibiae using micro-CT. Total bone volume (Figure 6A, 97.7% of vehicle) and trabecular bone volume fraction (Figure 6B, 99.7% of vehicle) were not affected by the combination treatment compared with vehicle. However, trabecular thickness (Figure 6C, 114.7% of vehicle) and trabecular separation (Figure 6D, 136.5% of vehicle) were increased by enzalutamide monotherapy, but not by the combination treatment when compared with vehicle. Conversely, a decreasing trend in trabecular number (Figure 6E, 93.1% of vehicle) and cross-sectional cortical bone area (Figure 6F, 89.9% of vehicle) was associated with the combination treatment. In addition, the combination treatment reduced cortical thickness (91.9% of vehicle) (Figure 6G), whereas bone specific surface (104.5% of vehicle) in cortical bone slightly increased (Figure 6H). Most changes associated with the combination treatment were considered minor i.e., less than a 10% change in the respective mean value of vehicle-treated mice. Altogether, these data indicate that concurrent treatment with radium-223 and enzalutamide did not markedly compromise bone health in the used preclinical setting.

## 3. Discussion

The development of mCRPC is often associated with AR pathway dysregulation which limits the efficacy of drugs targeting this pathway. Therefore, combination strategies enhancing the efficacy of AR-targeting agents in mCRPC are needed [17]. While enzalutamide and radium-223 are commonly used as single agent for treating patients with mCRPC, real-world data show that they are sometimes also used together, usually in a sequential way [18]. Both radium-223 and enzalutamide prolong the survival of men with mCRPC [2,4].

In this study, we demonstrate, for the first time, the synergistic and enhanced effects of radium-223 and enzalutamide combination treatment *in vitro* and *in vivo*, respectively. Enzalutamide in combination with radiation therapy has been shown to decrease the survival of AR-positive LNCaP cells *in vitro*, but the treatment had no effect on AR-negative PC-3 prostate cancer cells [19], suggesting that the enzalutamide-related radiosensitization in LNCaP cells is mediated via AR. Furthermore, it has been demonstrated in several *in vitro* and *in vivo* prostate cancer models, including LNCaP expressing mutated or wild type AR, that radiation therapy can upregulate AR signaling in CRPC leading to radioresistance [20]. We have previously shown that radium-223 monotherapy exhibits a dual targeting mode-of-action by inducing DNA double-strand breaks in both cancer cells and bone cells, thereby inhibiting tumor-induced pathologic bone formation in the LuCaP 58 xenograft model [21]. As both androgen deprivation therapy and AR inhibitors can suppress DNA damage repair genes [22], concurrent treatment with enzalutamide could make cancer cells more vulnerable to radium-223-induced DNA double-strand breaks. Notably, 32 genes associated with DNA damage repair have been shown to be androgen-regulated in LNCaP cells [23].

To support the hypothesis of enzalutamide-related radiosensitization behind the synergistic antiproliferative effects of radium-223 and enzalutamide, we studied the expression of genes associated with DNA damage repair in R1881-stimulated LNCaP cells treated with or without enzalutamide *in vitro*. In addition to the GSEA that revealed the downregulation of pathways related to DNA damage repair, the expression of several relevant genes was markedly downregulated by enzalutamide. The expression of Claspin (*CLSPN*), for instance, a key protein in checkpoint mediated cell cycle arrest in response to DNA damage induced by ionizing and UV radiation whose downregulation inhibits CHK1 activation [24], was markedly downregulated. Consequently, Claspin interacts with BRCA1 [25], and its gene expression itself was downregulated by 50% upon enzalutamide treatment. Another example is DNA replication ATP-dependent helicase/nuclease DNA2 that is recruited by Bloom helicase (BLM) to DNA double-strand breaks and is involved in processing of 5′-ssDNA ends [26], was also downregulated after enzalutamide treatment. Thus, downregulation of DNA damage repair genes by enzalutamide could radiosensitize LNCaP cells to radium-223, and, therefore, be one plausible mechanism explaining the observed synergistic/enhanced efficacy of radium-223 and enzalutamide combination treatment in the LNCaP model.

When enzalutamide is combined with radium-223, their effects in the bone microenvironment are prominent. Signs of enzalutamide resistance have been found in bone metastases of enzalutamide-treated patients, and the response to enzalutamide in the bone microenvironment is suggested to be dependent on the patient’s androgen-signaling status [27]. Here, in the intratibial LNCaP xenograft model, the combination of radium-223 and enzalutamide resulted in decreased serum PSA levels and areas of tumor-induced abnormal bone, while either radium-223 or enzalutamide alone did not decrease serum PSA. Previously, we have shown that radium-223 at the same dose level as in this present study has limited antitumor efficacy on PSA in the intratibial LNCaP model [21]. However, here, the end point of PSA was evaluated 2 weeks earlier than in the previous study, which could explain why radium-223 (or enzalutamide) had no marked effect on the PSA concentration this time. Furthermore, the second dose of radium-223 was given only one day before the sacrifice and the last PSA sampling, thus, its potential effects on antitumor efficacy were probably not observable at this point. In another previous preclinical study using this model, radium-223 in combination with abiraterone and prednisone did not show additive/synergistic antitumor effects [28]. This lack of efficacy might be explained by abiraterone/prednisone-mediated reduction of radium-223 uptake to abnormal bone. Importantly, enzalutamide had no effect on radium-223 uptake in our study.

Radium-223 in combination with enzalutamide had a significant impact on bone turnover as indicated by decreased levels of the bone formation marker PINP and the bone resorption marker CTX-I. This is in line with our previous studies, where we have shown that radium-223 decreased PINP levels in serum, reflecting the inhibition of pathological bone changes in the LNCaP model [21]. As monotherapy, radium-223 showed a decreasing trend on the PINP and CTX-I levels, but enzalutamide did not have any effect. In a prospective phase 2 trial, decreased levels of bone markers, including PINP, were associated with improved outcomes in mCRPC patients treated with the combination of radium-223 and enzalutamide, whereas no such impact was seen in patients treated with enzalutamide monotherapy [29].

We have previously reported that radium-223 decreased the tumor-induced bone formation by inhibiting the bone turnover in LuCaP 58 tumor-bearing tibiae in mice [21]. In this study, we observed a decrease in the number of osteoblasts and abnormal bone formation upon radium-223 monotherapy, as well as in combination with enzalutamide. In mice, enzalutamide has been shown to increase the number of osteoblasts and osteoclasts in the trabecular bone of L4 vertebra, but not in tibiae [30]. In our study, concurrent treatment with enzalutamide did not impair the direct effects of radium-223 on osteoblasts, as indicated by similar numbers of osteoblasts in the non-tumor-bearing tibiae upon combination treatment or radium-223 monotherapy. In line with these results, radium-223 alone, and in combination with enzalutamide, resulted in a decrease in the trabecular and periosteal mineralizing surface, whereas enzalutamide monotherapy increased the trabecular mineralizing surface in the non-tumor-bearing tibiae.

Certain characteristics of the LNCaP model may complicate the interpretation of bone metabolism marker results. First, when evaluating systemic PINP and CTX-I levels, one should note that both the treatment-induced and cancer-induced changes in bone turnover are reflected by these bone markers. Our bone marker results suggested that radium-223 in combination with enzalutamide abrogates the bone turnover-inducing effect of enzalutamide. This is supported by the observed increase in the mineralizing surface parameters of enzalutamide-treated tibiae. Moreover, during the treatment, the normal growth of bones in young mice was slowing down, which also affected the bone marker levels and further complicated the interpretation of the results.

The effects of radium-223 and enzalutamide on normal bone microarchitecture in non-tumor-bearing bones were evaluated using micro-CT. We observed no major effects compromising bone structure or microarchitecture upon the combination treatment; however, specific changes in trabecular and cortical bone were associated with the combination treatment. Whether these changes contribute to the risk of non-pathologic fractures remains to be studied. Recent studies suggest that radium-223 in combination with enzalutamide prolongs progression-free survival without increasing the risk of fractures in patients with bone-metastatic CRPC [14,31]. In the first interim safety analysis of the ongoing PEACE III phase 3 trial, no bone fractures were reported in either of the treatment arms when patients were supplemented with denosumab or zoledronic acid [15], whereas a recent safety update with a longer follow-up period showed only a few fractures in these groups [16]. Similar results were also recently found in a retrospective analysis of mCRPC patients treated with radium-223 and abiraterone [32].

In conclusion, this study demonstrated the enhanced antitumor efficacy of the radium-223 and enzalutamide combination *in vitro* and in an *in vivo* xenograft model mimicking prostate cancer metastasized to bone. The combination treatment also prevented the tumor-induced pathological effects on bone, i.e., it reduced the number of osteoblasts and abnormal bone formation in the tumor-bearing bone. Unlike the previously evaluated combination treatment with radium-223, abiraterone and prednisone, the concurrent enzalutamide treatment with radium-223 did not affect radium-223 uptake to bone. This could potentially explain the observed enhanced antitumor effects of this combination. Our findings support the ongoing phase 3 clinical trial, PEACE III (NCT02194842) in which the combination of radium-223 and enzalutamide is being investigated in patients with CRPC metastasized to bone.

## 4. Materials and Methods

### 4.1. In Vitro Cell Viability Assay

The cell viability of androgen-responsive LNCaP human prostate cancer cells was measured using the CellTiter-Glo^®^ assay (Promega, Madison, WI, USA) after 6 days of exposure to radium-223 and the antiandrogen enzalutamide. Isobolograms and combination indexes were calculated according to Chou-Talalay [33], with combination index <0.7 defined as synergistic effect. EC_50_, EC_70_, and EC_90_ values were calculated for each individual combination data point, and the isobolograms were generated. These results were confirmed in the second experiment. The assay is described in more detail in the Supplementary Materials.

### 4.2. In Vitro Androgen Stimulation Assay and RNA Sequencing

LNCaP cells were stimulated with the synthetic androgen R1881 at a concentration of 1 nM after 2 days of starvation in medium supplemented with 10% charcoal-stripped fetal bovine serum FBS. Enzalutamide was added at a final concentration of 2 µM, and the cells were harvested 22 h post-treatment.

Cells were lyzed and RNA was isolated using RNeasy columns with on-column DNA digestion, as described by the manufacturer (Qiagen, Hilden, Germany). RNA integrity was measured using an Agilent 2100 Bioanalyzer (Agilent, Santa Clara, CA, USA), and samples with RNA integrity number (RIN) values above eight were further processed. RNA library preparation was performed after mRNA purification using poly-T beads, as described by the manufacturer (TruSeq Stranded mRNA Kit; Illumina, San Diego, CA, USA). Ten and five biological replicates for the combination and R1881 treatment, respectively, were sequenced on a HiSeq2500 device via single-end, 50 base-pair reads with an average depth of 21 million reads per sample (Illumina, HiSeq2500 HTv4, SR, dual-indexing, 50 cycles). The sequencing data is publicly available through the Gene Expression Omnibus (GEO) database (accession number GSE220097).

FASTQ reads were mapped via STAR aligner to the human genome GRCh38 and quantified with featureCounts from the Subread package [34]. In each treatment group, expression was summarized as median count per million values (CPM) by gene. Gene set enrichment analysis (GSEA) [35] was performed using the Fast Gene Set Enrichment (fgsea) package version 1.22.0 [36] on the ratio of median CPM values from the combination treatment versus single R1881 treatment. GSEA was restricted to Reactome pathways [37] in the Molecular Signatures Database version 2022.1 [38] with at least 15 genes and at most 500. Differentially regulated pathways were defined as having adjusted *p*-values lower than 0.05.

### 4.3. Intratibial LNCaP Xenograft Model

**Figure 7:**
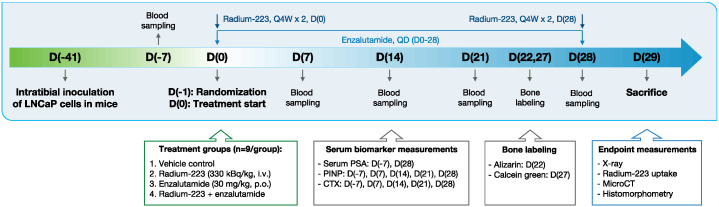
The study outline and timeline of the intratibial LNCaP model. Six weeks after inoculation, the mice were stratified to treatment groups and treated with vehicle, radium-223 (330 kBq/kg, Q4W × 2, i.v., on days 0 and 28), enzalutamide (30 mg/kg, QD × 28, p.o.), or a combination of radium-223 and enzalutamide for 28 days. D(number) indicates days from treatment start.

LNCaP cells (2 × 10^6^ in 20 µL of PBS), which secrete PSA [39], were inoculated into the bone marrow cavity of the right proximal tibia of 5–7 weeks old male NOD.scid mice (NOD.CB17/Prkdcscid/scid/Rj, Envigo, Horst, The Netherlands). When inoculated intratibially, LNCaP cells form mixed osteoblastic/osteolytic lesions typically found in patients with metastatic prostate cancer [21]. Six weeks after the inoculation, stratification was performed using serum PSA (*n* = 9/group). The groups were treated with: (I) vehicle (28 mmol/L sodium citrate every four weeks with two doses in total (Q4W × 2), i.v. and peanut oil with 5% benzyl benzoate once daily (QD) p.o.), (II) radium-223 (330 kBq/kg, Q4W × 2, i.v.), (III) enzalutamide (30 mg/kg, QD, p.o.) or (IV) combination of radium-223 (330 kBq/kg, Q4W × 2, i.v.) and enzalutamide (30 mg/kg, QD, p.o.) for 28 days (Figure 7). Animal experiments were approved by the Animal Experiment Board in Finland (license number: ESAVI-2331-04 10 07-2017) and followed the guidelines of the European Union directive 2010/63/EU. Additional procedures are described in the Supplementary Materials.

### 4.4. PSA and Biochemical Markers of Bone Turnover

Blood samples (100–200 μL) were collected after six hours of fasting from the saphenous vein seven days before randomization and once weekly after the initiation of treatments. At sacrifice, blood samples were obtained through a cardiac puncture. The samples were collected into Microvette 100 Z & 200 Z-Gel tubes (Sarstedt Ag & Co, Nümbrecht, Germany) and were gently inverted. The blood was allowed to clot at room temperature for 30–60 min followed by a centrifugation at 10,000× *g* at room temperature for 5 min. The serum samples were stored at −80 °C. Serum PSA was measured at two time points, seven days before treatment start and at the end of the study (28 days post-treatment). Levels of the bone formation marker PINP and the bone resorption marker CTX-I were measured seven days before the initiation of treatments and on days 7, 14, 21, and 28. The markers were analyzed using the Human Kallikrein 3/PSA Quantikine® enzyme-linked immunosorbent assay (ELISA) kit (R&D Systems, Minneapolis, MN, USA), and Rat/Mouse PINP and RatLaps® CTX-I ELISA kits (both from IDS Ltd, Boldon, UK). These kits utilize quantitative sandwich or competitive ELISA techniques with peroxidase-linked antibodies specific for human PSA, PINP or CTX-I, and chromogenic (tetramethylbenzidine) color detection. The measurements were done according to the manufacturers’ instructions. Serum samples for PSA (45 µL), PINP (5 µL) and CTX-I (15 µL) measurements were added to pre-coated microplates. Equivalent volumes of assay standards and controls were used. For quantification, absorbance was measured at 450 nm using a VICTOR2 Multilabel Counter (PerkinElmer, Waltham, MA, USA).

### 4.5. Radiography of Tumor-Bearing Tibiae

Tumor-bearing tibiae were imaged using a Faxitron Specimen Radiographic System MX-20 D12 (Faxitron Corp., Wheeling, IL, USA) and Faxitron Dicom software v3.0. Tumor-induced abnormal bone area was determined from the X-ray images using MetaMorph image analysis software (Molecular Devices LLC, Sunnyvale, CA, USA).

### 4.6. Ex Vivo Analyses

At sacrifice, hind limbs were collected, and bone volume, cross-sectional dimensions, and bone structure of non-tumor-bearing tibiae were quantified using a SkyScan 1276 high-resolution micro-CT scanner (Bruker, Kontich, Belgium). Bone formation and cellular characteristics were analyzed by bone histomorphometry using an OsteoMeasure7 histomorphometry system (OsteoMetrics, Atlanta, GA, USA). Micro-CT and bone histomorphometry parameters are listed in Tables S1 and S2, respectively. To evaluate radium-223 uptake in bone, the radioactivity of tumor-bearing and non-tumor-bearing tibiae was measured using an automatic gamma counter (Hidex, Turku, Finland). Tumor-bearing tibia histology sections were stained with H&E and analyzed using Pannoramic 250 Flash and Pannoramic 1000 slide scanners (3DHISTECH Ltd., Budapest, Hungary). The methods are described in detail in the Supplementary Materials.

### 4.7. Statistical Analyses

Statistical analyses were performed using the R statistical software v3.6.3 [40]. Longitudinal biomarker data (PSA, PINP, CTX-I) were log-transformed and analyzed using mixed models and model contrasts. For PSA data, the values at the end of the study relative to baseline were analyzed using ANOVA, and the pairwise comparisons using ANOVA contrasts. For PINP and CTX-I, the data were modeled using a linear mixed-effects model and the comparisons were performed using model contrasts. For *ex vivo* radiography analysis, the data were analyzed using ANOVA after square root transformation. Micro-CT and histomorphometry data were analyzed using ANOVA followed by contrasts, or Kruskal-Wallis test followed by Dunn’s test. Kruskal-Wallis test and pairwise comparisons using Dunn’s test were applied for histology analyses. Radium-223 uptake data were analyzed using Welch’s *t*-test. All statistical tests used were two-sided. The obtained *p*-values were adjusted for all analyses.

## Data Availability

The data presented in this study are openly available in the Gene Expression Omnibus database (accession number GSE220097), in the article and in its online Supplementary Materials.

## Supplementary Materials

The supporting information can be downloaded at: https://www.mdpi.com/article/10.3390/ijms24032189/s1. References [41,42,43,44,45,46] are cited in the Supplementary Materials.
