# Supplementary material for: Enhanced Antitumor Efficacy of Radium-223 and Enzalutamide in the Intratibial LNCaP Prostate Cancer Model
Source: Int J Mol Sci. 2023 Jan 22;24(3):2189. doi: 10.3390/ijms24032189 (PMC9916479; doi:10.3390/ijms24032189)
Supplement: Supplementary file 1 [file ijms-24-02189-s001.zip › ijms-2054677-supplementary.pdf]

SUPPLEMENTARY MATERIALS

Enhanced antitumor efficacy of radium-223 and enzalutamide in the intratibial LNCaP prostate cancer model

Mari I. Suominen[1], Matias Knuuttila[2], Christoph A. Schatz[3], Andreas Schlicker[3], Jukka Vääräniemi[1], Birgitta Sjöholm[2], Esa Alhoniemi[4], Bernard Haendler[3], Dominik Mumberg[3], Sanna-Maria Käkönen[2,5], Arne Scholz[3]

[1]Pharmatest Services Ltd., Turku, Finland;

[2]Aurexel Life Sciences Ltd., Askainen, Finland;

[3]Bayer AG, Research & Development, Pharmaceuticals, Berlin, Germany ;

[4]Inoi Oy, Turku, Finland

[5]Institute of Biomedicine, University of Turku, Turku, Finland

SUPPLEMENTARY METHODS

Compounds

Radium-223 dichloride (radium-223) was synthesized at Bayer AG. For *in vivo* studies, radium-223 diluted with 28 mmol/L sodium citrate to form a solution with a radium-223 concentration of 66 kBq/mL. Enzalutamide (MedChemExpress, Sollentuna, Sweden) was dissolved in saline with 10% dimethyl sulfoxide (DMSO), 40% polyethylene glycol 300 (PEG300) and 5% Tween80 to reach a final concentration of 2.5 mg/mL.

Cell culture

LNCaP cells human prostate cancer cells (CRL-1740™, ATCC) were cultured in standard cell culture conditions and authenticated using short tandem repeat analysis (GenePrint10 system, Promega, Madison, WI, USA) at the Institute for Molecular Medicine Finland (FIMM, Helsinki, Finland) in June 2014. The cells were tested negative for mycoplasma using an IDEXX PCR test and murine pathogens by IDEXX Laboratories Inc (Ludwigsburg, Germany).

***In vitro* cell viability assay**

LNCaP cells were cultured in standard cell culture conditions (+37°C, 5% CO_2) and seeded with a density of 1000 cells/well onto CulturPlate-384 well plates (#6007689, PerkinElmer, Waltham, MA, USA) in assay medium consisting of RPMI-1640 phenol-red free medium (FG 1215, Biochrom, Cambridge, UK) and 10% charcoal-stripped fetal bovine serum (cFBS). Next day, after adding fresh assay medium containing 0.3 nM R1881, radium-223 (0.04-20kBq/mL) or enzalutamide (0.04-20 µM) were added to the 384-well plates using the D300e digital dispenser (Tecan, Männedorf,

Switzerland). The cells were incubated for 6 days before the cell viability was determined using CellTiter-Glo® (CTG) assay (#G7573, Promega, Madison, WI, USA) by adding 30 µL of CTG reagent directly to the cells. The plates were put on a shaker (400 rpm) for 2 minutes and incubated for 8 minutes at RT. Luminescence was measured using a PHERAstar® FSX plate reader (BMG LABTECH, Ortenberg, Germany).

Intratibial LNCaP model

Male non-obese diabetic (NOD) mice (25 g at the beginning of the study) with severe combined immunodeficiency (scid) (NOD.scid; NOD.CB17/*Prkdc*$^{scid/scid}$/Rj, Envigo, Horst, Netherlands) were earmarked, housed in individually ventilated cages (IVC), fed an irradiated soy free diet (Teklad Global Diets 2916, Envigo, Madison, WI, USA) and autoclaved tap water *ad libitum*. The minimum quarantine and acclimatization period for the mice was 5 days. For intratibial inoculations and imaging, the mice were anesthetized with inhalation of isoflurane (IsoFlo vet 100%, Zoetis Finland Oy, Helsinki, Finland). Analgesia was provided with buprenorphine (Temgesic 0.3 mg/mL, Indivior Europe Ltd, Dublin, Ireland): 0.1 mg/kg, subcutaneously (s.c.) before and 0.02 mg/mL in drinking water for 2 days after the intratibial inoculation. All inoculations were done using two separate cell batches in one day and the sufficient viability of the cells was confirmed before inoculation. The animals were weighed twice a week and observed daily to monitor the progression of disease. Appearance of any clinical signs were recorded on follow-up forms. First PSA measurements were performed six weeks after the inoculation. At the end of the study, the mice were sacrificed by CO_2 followed by cervical dislocation. At necropsy, all macroscopic findings were recorded.

The bones were collected into 40% cold ethanol in separate liquid scintillation vials for measuring radium-223 uptake.

Micro-CT analysis

Micro-CT measurements *ex vivo* for bone volume, cross-sectional dimensions and microarchitecture in the non-tumor-bearing tibiae were quantified using a SkyScan 1276 high-resolution micro-CT scanner (Bruker, Kontich, Belgium) [1-3]. Two separate volume of interests (VOI) were analyzed in metaphyseal trabecular bone and one VOI in diaphyseal cortical bone. The reported micro-CT parameters are summarized in **Supplementary Table 1**.

Bone labeling and histomorphometry

For measuring dynamic histomorphometry parameters, bone was labeled by an injection of fluorescence dye twice during the treatment period of the study. Solid alizarin (#A3882; Sigma-Aldrich, St. Louis, MO, USA) and solid calcein green (#C0875; Sigma-Aldrich) were dissolved in 2% sodium bicarbonate and 0.9% NaCl, respectively. Subsequently, the dosing solutions containing 15 mg/mL of alizarin and 2 mg/mL of calcein green were sterile-filtered. Bone labeling was performed with alizarin (30 mg/kg, s.c.) seven days before sacrifice and with calcein green (10 mg/kg, s.c.) two days before sacrifice. The mice in the pre-treatment group were not injected.

Static and dynamic histomorphometry parameters were analyzed using an OsteoMeasure7 histomorphometry system (OsteoMetrics, Atlanta, GA, USA) in axial skeleton [4, 5]. Bone samples were dehydrated in ethanol, defatted in xylene, and

embedded in methyl methacrylate-based plastic. After the embedding, longitudinal sections were obtained from a standardized site of proximal tibia using a fully motorized rotary microtome and a tungsten-carbide knife. Static trabecular bone parameters were determined in two 4-μm-thick sections stained in Masson-Goldner's trichrome and dynamic trabecular bone parameters in two unstained 8-μm-thick sections. Cross-sectional cylinders were prepared from a standardized site of tibial shaft in a transverse plane using a linear precision saw and a diamond blade. Both static and dynamic cortical bone parameters were determined in an unstained 200-μm-thick cylinder. All parameters were analyzed following the guidelines of the American Society for Bone and Mineral Research (ASBMR) [6], including the reported parameters summarized in **Supplementary Table 2**.

Histology

Tumor-bearing tibiae were cut from the diaphysis to save bone samples for cortical histomorphometry. The proximal end of the tibia was processed to plastic. Longitudinal 4-μm sections were obtained and stained with Masson-Goldner trichrome like previously described for histomorphometry. The slides were scanned using Pannoramic 250 Flash and Pannoramic 1000 slide scanners (3DHISTECH Ltd, Budapest, Hungary). Tumor area, cortical and trabecular bone areas, and fibrotic and necrotic areas were analyzed from both a standardized area (encompassing 5 mm from the articular surface) and the whole section using CaseViewer software version 2.3 (3DHISTECH Ltd). Tumor, fibrotic and necrotic areas were drawn manually, and bone area was defined by color threshold.

SUPPLEMENTARY REFERENCES

1. Bouxsein ML, Boyd SK, Christiansen BA, et al. Guidelines for assessment of bone microstructure in rodents using micro-computed tomography. *J Bone Miner Res* 2010;25(7):1468-86.

2. van 't Hof RJ. Analysis of bone architecture in rodents using microcomputed tomography. *Methods Mol Biol* 2012;816:461-76.

3. Engelke K, Prevrhal S, Genant HK. Macro- and microimaging of bone architecture. In: John P. Bilezikian LGR, T. John Martin, (ed). *Principles of bone biology*. San Diego, CA, USA: Academic Press; **2008**, 1905-1942.

4. Dempster DW. Histomorphometric analysis of bone remodeling. In: John P. Bilezikian LGR, T. John Martin, (ed). *Principles of bone biology*. San Diego, CA, USA: Academic Press, San Diego; **2008**, 447-463.

5. Erben RG, Glosmann M. Histomorphometry in rodents. *Methods Mol Biol* 2012;816:279-303.

6. Dempster DW, Compston JE, Drezner MK, et al. Standardized nomenclature, symbols, and units for bone histomorphometry: a 2012 update of the report of the ASBMR Histomorphometry Nomenclature Committee. *J Bone Miner Res* 2013;28(1):2-17.

SUPPLEMENTARY TABLES

Supplementary Table S1: Micro-CT parameters

**Bone**	**Parameter**	**Standard unit**
Total	Bone volume	BV (mm^3)
Trabecular	Bone volume fraction	BV/TV (%)
	Thickness	Tb.Th (μm)
	Separation	Tb.Sp (μm)
	Number	Tb.N (mm^{-1})
Cortical	Cross-sectional bone area	Ct.Ar (mm^2)
	Specific bone surface	BS/BV (%)
	Thickness	Ct.Th (μm)

Ar, area; BV, bone volume; BS, bone surface; Ct, cortical; N, number; Sp, separation; Tb, trabecular; Th, thickness; TV, tissue volume.

Supplementary Table S2: Dynamic and static histomorphometry parameters

Method	Parameter	Tibia	Standard unit
Dynamic	Trabecular mineralizing surface per bone surface	non-tumor-bearing	MS/BS (%)
	Periosteal mineralizing surface per bone surface	non-tumor-bearing	MS/BS (%)
	Endocortical mineralizing surface per bone surface	non-tumor-bearing	MS/BS (%)
Static	Osteoblast number in ratio to bone perimeter	non-tumor-bearing	N.Ob/Bp (mm^{-1})
	Osteoclast number in ratio to bone perimeter	non-tumor-bearing	N.Oc/Bp (mm^{-1})
	Osteoblast number in ratio to bone perimeter	tumor-bearing	N.Ob/Bp (mm^{-1})
	Osteoclast number in ratio to bone perimeter	tumor-bearing	N.Oc/Bp (mm^{-1})

Bp, bone perimeter; BS, bone surface; MS, mineralizing surface; N.Ob, osteoblast number; N.Oc, osteoclast number
